# Neuroticism is Associated With Greater Affective Variability at High Levels of Momentary Affective Well‐Being, but With Lower Affective Variability at Low Levels of Momentary Affective Well‐Being

**DOI:** 10.1111/jopy.12972

**Published:** 2024-09-06

**Authors:** Mario Wenzel, Whitney R. Ringwald, Aleksandra Kaurin, Oliver Tüscher, Thomas Kubiak, Aidan G. C. Wright

**Affiliations:** ^1^ Department of Psychology Johannes Gutenberg‐Universität Mainz Mainz Germany; ^2^ Department of Psychology University of Minnesota Minnesota USA; ^3^ Department of Psychology Bergische Universität Wuppertal Wuppertal Germany; ^4^ Leibniz Institute for Resilience Research Mainz Germany; ^5^ Department of Psychiatry and Psychotherapy University Medical Center Mainz Mainz Germany; ^6^ Institute of Molecular Biology (IMB) Mainz Germany; ^7^ Department of Psychology University of Michigan Ann Arbor Michigan USA; ^8^ Eisenberg Family Depression Center University of Michigan Ann Arbor Michigan USA

**Keywords:** ambulatory assessment, neuroticism, state–trait association, variability

## Abstract

**Objective:**

Research challenged the notion that neuroticism correlates with affective variability, suggesting that it may result from statistical artifacts due to the non‐normal distribution of negative affect. We aim to advance this line of research by (a) introducing affect balance as a normally distributed measure of affective well‐being and (b) examining current affect balance as a moderator of the relationship between neuroticism and affect balance variability.

**Method:**

We meta‐analyzed the results of 14 ambulatory assessment datasets (*N* = 2389 participants, *N* = 174,423 observations).

**Result**s**:**

We found that while the associations between the mean and affective variability were large for negative affect, they were much smaller for affect balance. Moreover, the association between neuroticism and variability in negative affect was very small, yet medium‐sized for affect balance. Importantly, the latter association depended on current affect levels: Participants high relative to low in neuroticism showed *stronger* subsequent fluctuations in affect balance when currently feeling better than usual, but *weaker* subsequent fluctuations in (and thus more persistent) affect balance when currently feeling worse than usual.

**Conclusion:**

Increased variability should not be seen as a bad sign but may be a sign that an affective system is changing, which may be adaptive or maladaptive for an individual, depending on the initial state of the system.

## 1 Introduction

Neuroticism has been conceptualized as a relatively stable tendency to respond to situations characterized by uncertainty and threat with negative affective states such as anger, anxiety, and sadness (Costa and McCrae 1992). In the past, it has been linked to indicators of mental (Jeronimus et al. 2016; Kotov et al. 2010; Malouff, Thorsteinsson, and Schutte 2005) and physical health (Charles et al. 2008; Goodwin, Cox, and Clara 2006). Thus, given the health significance of neuroticism (Lahey 2009), research efforts to understand how neuroticism manifests in individuals' daily lives are critical. A first step is to determine how best to characterize affective experiences in everyday life and how they relate to neuroticism. To this end, research has increasingly used ambulatory assessment methods, in which neuroticism is assessed at baseline and affective experiences are captured over the following week(s) through intensive, repeated sampling in daily life. In these studies, participants are typically asked to report their current affect once or several times a day. Each participant then has a distribution of affect based on these repeated assessments, which can vary widely between individuals. Statistical indices can then be used to describe these individual distributions and, thus, examine their interindividual differences (Papoulis and Pillai 2002). The most widely used measures in affective research are the mean and the variance/standard deviation, where the intraindividual mean (*iM*) reflects the affective level or location and the intraindividual standard deviation (*iSD*) captures the affective variability or scale.

Importantly, there is likely a reason behind the ups and downs of emotions in our daily experiences and it is plausible to assume that fluctuations in affective states aid monitoring and protecting people's needs, goals, and overall well‐being (Panksepp 2012). Theories on the role of emotions suggest that these fluctuations are beneficial and help people adapt to changes in their environment and the demands placed on them (e.g., Carver 2015; Panksepp 2012). Significant events trigger affective responses that lead to changes in people's behavioral and physiological regulatory systems. As a result, individuals may engage with or disengage from their environment. However, these theories also indicate that these fluctuations serve a purpose within defined limits. Regulatory mechanisms, such as the use of effective emotion regulation strategies, prevent them from becoming dysfunctional (e.g., Carver 2015; Kuppens and Verduyn 2017). If variations in affective experiences are essential for general well‐being, alterations in these variations may likely be connected to neuroticism.

And indeed, meta‐analytically, neuroticism has been linked to both increased levels of negative affect, $\bar{r}$ = 0.53 (Fleeson and Gallagher 2009) and $\bar{r}$ = 0.36 (Kalokerinos et al. 2020), but also to increased variability in negative affect, $\bar{r}$ = 0.18 (Houben, Van Den Noortgate, and Kuppens 2015) and $\bar{r}$ = 0.28 (Kalokerinos et al. 2020). However, there are two issues associated with this line of research. First, data from a recent study suggest that the link between neuroticism and affective variability may be due to a statistical artifact (Kalokerinos et al. 2020). When accounting for a mean‐adjusted version of *iSD*, instead of *iSD* (Mestdagh et al. 2018), the authors found a non‐significant association between neuroticism and affective variability that was very close to zero, $\bar{r}$ = 0.05. Second, meta‐analytic research also shows that neuroticism is significantly associated not only with increased variability and instability in negative affect (Houben, Van Den Noortgate, and Kuppens 2015), but also with more persistent negative affect, that is, negative affect inertia (Houben, Van Den Noortgate, and Kuppens 2015). Given that inertia has been viewed as resistance to change and thus reflects stability in affective states, it seems paradoxical that individuals high in neuroticism should experience affect as both more stable and more unstable than individuals low in neuroticism. In this paper, we advance the discussion of these two issues by introducing affect balance as a measure well suited for studying affective variability and its relationship to personality traits such as neuroticism, and by examining whether the relationship between neuroticism and affective variability depends on prior levels of affect, potentially resolving the stability–instability paradox.

### 1.1 Relation Between Neuroticism and Variability in Negative Affect as Captured via the *iSD*

Over the past two decades, research using ambulatory assessment has demonstrated a well‐established and replicable association between neuroticism and greater variability in negative affect as captured by the *iSD*: For example, a meta‐analysis of 61 effect sizes reported an average association between neuroticism and variability in negative affect of $\bar{r}$ = 0.18 (Houben, Van Den Noortgate, and Kuppens 2015), indicating that individuals high versus low in neuroticism reported greater variability in their negative emotions. These findings were replicated in the recent meta‐analysis by Kalokerinos et al. (2020), which demonstrated a medium‐to‐large average association between neuroticism and greater variability in negative affect of $\bar{r}$ = 0.28 across 11 ambulatory assessment datasets.

However, recent research has cast doubt on the importance of affective variability, as captured by the *iSD*, for neuroticism. Specifically, it has been argued that the positive relationship may be due to a non‐linear dependence between the *iM* and the *iSD* (Baird, Le, and Lucas 2006; Eid and Diener 1999; Kalokerinos et al. 2020), which should be controlled for when examining the relationship between neuroticism and affective variability as captured by *iSD*. One putative reason for this non‐linear confound is the use of bounded scales (Mestdagh et al. 2018). This concern is best illustrated by negative affect, which is not normally distributed in everyday life, but tends to be right‐skewed (e.g., Kalokerinos et al. 2020), and especially so in non‐clinical samples. If the boundary were the problem, then the range of possible variability values would be different depending on the mean.^1^

### 1.2 Research Goal #1: Introducing Affect Balance as an Affect Measure for the Relation Between Neuroticism and Affective Variability

To control for this confound, Mestdagh et al. (2018) proposed the relative *iSD* (*iRSD*), which is computed by dividing a given *iSD* by the maximum *iSD* at a given *iM*, resulting in a possible range of 0–1. Using this *iRSD* instead of the *iSD*, Kalokerinos et al. (2020) found that the mean association between neuroticism and negative affect variability decreased from $\bar{r}$ = 0.28 to a non‐significant $\bar{r}$ = 0.05. These results suggest that the relationship between neuroticism and negative affect variability may be the result of confounding with the *iM*.

However, there are statistical and conceptual issues with the *iRSD*. We briefly summarize the issues here and present a more detailed critique in the supplementary material (https://osf.io/wtasq?view_only=d97ddf7e9d29430f9c951a1daccca02f). First, prior research has shown that the *iRSD* does not properly correct for the confound between the *iSD* and *iM*, such that in about half of the datasets analyzed there, the *iM* ~ *iSD* confound was not eliminated by the use of the *iRSD*, and in about one‐third it was reversed (Wenzel and Kubiak 2020). Second, the *iRSD* produced statistical artifacts in the form of an inverse confound that was highly related to the relationship of interest, the relationship between neuroticism and affective variability: Not only did differences in the *iM* ~ *iRSD* associations fully explain differences in the Neuroticism~*iRSD* relationship (Wenzel and Kubiak 2020), but they were also highly correlated with variability in the Neuroticism–*iRSD* relationship, *r* = 0.86 (Wenzel et al. 2023). Importantly, this was not the case when *iSD* was used instead of *iRSD*. Third, only the *iSD*, but not the *iRSD*, showed convergent validity with another measure of negative affect variability. Fourth, we argue in this paper that there is also a conceptual problem with the *iRSD*. The *iRSD* tries to solve the *iM* ~ *iSD* confound by standardizing the *iSD* to the maximum *iSD* for a given *iM*. This means that very small *iSDs* at the limits of the scale can get very large *iRSDs*, but a theoretical reason beyond statistical confounding for the decision to give stronger weights to values closer to the bounds is not provided by Mestdagh et al. (2018). Finally, the skewed distribution of negative affect is a problem not only for the *iSD* but also for the *iM*, as the calculation of the mean depends on symmetric distributions (e.g., normal), making it sensitive to outliers. Thus, Ringwald and Wright (2022) recently argued that the median or mode of negative affect may be a better measure of central tendency because these indices are less sensitive to skew.

Taken together, the problem may not be that the *iSD* is constrained by the mean, as suggested by Kalokerinos et al. (2020), but that the distribution of negative affect in daily life is highly skewed. Given these issues, we, therefore, do not recommend using the *iRSD* because of its statistical and conceptual problems. Instead, we recommend using the *iSD* and controlling for confounding by including an affective level measure as a control variable that is less confounded with the *iSD* to avoid the problem of multicollinearity. However, we still have to deal with the highly skewed distribution of negative affect when using the *iM* as a control variable. In the present research, we propose to also include positive affective states such as happiness and relaxation in the affect measure (e.g., Tomitaka et al. 2016) and then calculate the *affect balance* as a measure of affective well‐being (Diener et al. 2010),^2^ as well as corresponding values of *iM* and the *iSD*. Affect balance is the difference between positive and negative affect and captures the preponderance of positive over negative affect, similar to bipolar negative/positive affect scales such as the Short Mood Scale (Wilhelm and Schoebi 2007). Calculating the *iM* and the *iSD* of affect balance rather than negative affect has two advantages. First, it might solve the issue of the highly skewed distribution of negative affect. Because positive affect is typically normally distributed in ambulatory assessment studies (Terluin, de Boer, and de Vet 2016), with means near the midpoint of the affect scale (e.g., Wenzel and Brose 2022), a composite measure such as affect balance should be less right‐skewed than negative affect, potentially resulting in less confounding between *iM* and *iSD* of affect balance. Second, affect balance has also a conceptual advantage over using negative affect, as it represents a more comprehensive measure of affective well‐being (also called hedonic or emotional well‐being, Park et al. 2023), which not only incorporates (low) levels of negative affect but also (high) levels of positive affect. While NA is a core aspect of neuroticism, previous research has shown that neuroticism is associated not only with significantly higher levels of negative affect (Hisler et al. 2020; Kalokerinos et al. 2020; Wenzel et al. 2023), but also with low levels of positive affect (e.g., Ng 2009). Thus, the experience of negative affect may differ in the presence and absence of positive affect, which can be captured by the affect balance.^3^ Consequently, the first research goal of the present study was to test whether affect balance is a suitable measure in the study of affective variability and its relation to neuroticism.

### 1.3 Research Goal #2: Solving the Stability–Instability Paradox

However, previous research has found that neuroticism is not only significantly associated with greater variability in negative affect (which is closely related to (greater) instability), but also with greater negative affect inertia (i.e., greater stability) (Houben, Van Den Noortgate, and Kuppens 2015). Negative affect inertia reflects the first‐order autocorrelation of negative affect, in which affect at *t* is regressed on prior affect at *t* − 1, thereby capturing the extent to which negative affect carries over from one moment to the next (Dejonckheere et al. 2019). The finding of both greater instability and greater stability (i.e., greater inertia) has been described as paradoxical, as individuals could not be described as having more stable and more unstable affect at the same time than other individuals (Bos, de Jonge, and Cox 2019; Koval et al. 2013). One explanation for this paradoxical finding is that the *iSD* captures both stability and instability. Previous research has shown that affective instability, that is, moment‐to‐moment fluctuations in affect, operationalized by intraindividual mean squared successive differences (*iMSSD*), is related to affect inertia as follows: $\text{iMSSD}=2\times {\mathrm{SD}}^{2}\times \left(1-\text{inertia}\right)$ (Jahng, Wood, and Trull 2008). Solving for *iSD* gives the following equation: $\mathrm{iSD}=\sqrt{\frac{\text{iMSSD}}{2*\left(1-\text{inertia}\right)}}$. Thus, the *iSD* (i.e., affective variability) is large when the *iMSSD* (i.e., affective instability) is large, so the person experiences strong moment‐to‐moment fluctuations in affect, and when inertia (i.e., affective stability in the sense of resistance to change) is large. Please note that inertia can also capture changes in affect but these resolve slowly over a longer period of time (Nelson et al. 2020).

In the present research, however, we propose an alternative explanation for why individuals may have both more stable and more unstable affect at the same time. We propose that the relationship between neuroticism and affective variability depends on how individuals feel in the moment. Specifically, we propose that if an individual is in a state or episode of high affective well‐being, fluctuations would mainly indicate decreasing levels of affective well‐being, and thus variability could be viewed as maladaptive. However, if an individual in a state or episode of low affective well‐being, fluctuations would mainly indicate improved affective well‐being and thus variability could be seen as adaptive. This assumption is related to the notion in research on early warning signals for the transition to depression that increased variance in affect is a sign that the system is destabilizing and thus transitioning into (as signaled by increased variance in negative affect) or out of (as signaled by increased variance in positive affect, van de Leemput et al. 2014) depression.

However, there is currently no evidence to support this notion with respect to neuroticism. The only relevant evidence focuses on overall levels of affect as a moderator of the relationship between depressive symptoms and variability. One study found that variability in positive affect was associated with more depressive symptoms at high levels of positive affect, but with fewer depressive symptoms at low levels (Maher et al. 2018). Another study reported that greater variability in negative affect was significantly associated with fewer social anxiety symptoms for high levels of negative affect but more symptoms for low levels (Farmer and Kashdan 2014). Finally, a recent study reanalyzed seven ambulatory assessment datasets and did not replicate the two‐way interaction for positive affect but for negative affect (Maciejewski et al. 2023). However, all three of these studies used overall affect levels (i.e., the *iM*) at the between‐person level, whereas we propose that using momentary affect at the within‐person level is a better test of our hypothesis. When using overall affect levels at the between‐person level one is testing a between‐person hypothesis, that is, does the neuroticism‐variability association depend on how an individual typically feels? However, we are interested in within‐person changes, that is, does the neuroticism‐variability association depend on how an individual is currently feeling? To test this, we assessed momentary variability by the square root of the squared difference between two consecutive measurement occasions, reflecting moment‐to‐moment changes in affect.

### 1.4 The Present Study

The aim of the present study was to increment the current debate about the importance of affective variability underlying neuroticism. Specifically, we sought to examine the mean‐variability confound and hypothesized that the *iM* would be less strongly correlated with the *iSD* for affect balance than for negative affect (Hypothesis 1). Given the reduced confound, we further hypothesized that neuroticism would positively predict variability in affect balance over and above mean affect balance (Hypothesis 2). Finally, we examined whether the relationship between neuroticism and variability in affect balance would be moderated by current affect balance (Hypothesis 3). Specifically, we hypothesized that individuals high versus low in neuroticism would report greater variability in affect balance for high levels of affect balance (Hypothesis 3a), but less variability for low levels of affect balance (Hypothesis 3b). To test our hypotheses, we meta‐analyzed 14 ambulatory assessment datasets that used within‐day surveys. Seven of the datasets were also included in the meta‐analysis by Kalokerinos et al. (2020)^4^ and seven datasets were added by the authors of the present study.

## 2 Methods

### 2.1 Transparency and Openness

We affirm that we reported all manipulations and exclusions in the present research but not all measures because we secondary analyzed ambulatory assessment datasets that were collected for a different purpose. Consequently, the sample sizes of the individual datasets were not determined by the goals of the present research and none of the analyses presented here were planned before collecting the data or were pre‐registered and are, thus, exploratory. The data, analysis scripts, and the supplementary materials are made available on the Open Science Framework (https://osf.io/t56a4/?view_only=d97ddf7e9d29430f9c951a1daccca02f), whereas Dataset 14 comes from the project “STECCO – Starting Tertiary Education during the Corona Crisis: A Challenge and an Opportunity” (Sosin, Kramer, and Neubauer 2022) and can be accessed at the OSF project page (https://osf.io/bhq3p/). The datasets drawn from prior research can also be found on OSF (https://osf.io/gvfdx/).

### 2.2 Participants and Procedure

Participants’ characteristics can be found in the online supplement (Table S1 at https://osf.io/wtasq?view_only=d97ddf7e9d29430f9c951a1daccca02f). As in prior research, we excluded participants who completed less than one‐third of the observations (REDACTED). In total, our meta‐analysis is based on a total of *N* = 2389 participants with a total of *N* = 174,423 observations based on within‐day surveys from 14 ambulatory assessment datasets.^5^ For information regarding the procedure, we refer to the references denoted in Table S1. Descriptive statistics and the zero‐order correlations are detailed in Table 1.

**TABLE 1:** Mean, standard deviation, between‐person reliability, and zero‐order correlations of the study variables.

Measure	*M*	*SD*	Rel.	Stab.	1.	2.	3.	4.	5.	6.
1. Neuroticism	44.0	21.4	0.79^a^	—	—					
2. Mean positive affect	53.1	15.7	0.97^b^	0.84	**−0.35** ^**c**^ [−0.41, −0.29]	—				
3. Mean negative affect	15.1	12.4	0.98^b^	0.85	**0.40** [0.36, 0.44]	**−0.42** ^**c**^ [−0.52, −0.32]	—			
4. Mean affect balance	38.0	22.7	0.97^b^	0.83	**−0.44** [−0.49, −0.40]	**0.88** ^**c**^ [0.86, 0.90]	**−0.81** ^**c**^ [−0.85, −0.77]	—		
5. Variability in positive affect	15.1	5.1	0.85^b^	0.79	**0.15** [0.10, 0.19]	−0.06^**c**^ [−0.15, 0.03]	**0.11** ^**c**^ [0.03, 0.19]	**−0.09** ^**c**^ [−0.18, −0.01]	—	
6. Variability in negative affect	10.4	5.2	0.84^b^	0.71	**0.36** [0.32, 0.40]	**−0.25** ^**c**^ [−0.34, −0.16]	**0.59** ^**c**^ [0.53, 0.65]	**−0.48** ^**c**^ [−0.55, −0.41]	**0.58** ^**c**^ [0.52, 0.64]	—
7. Variability in affect balance	22.2	8.5	0.84^b^	0.76	**0.28** [0.24, 0.32]	**−0.13** ^**c**^ [−0.24, −0.03]	**0.36** ^**c**^ [0.27, 0.44]	**−0.27** ^**c**^ [−0.37, −0.18]	**0.93** [0.92, 0.94]	**0.83** ^**c**^ [0.79, 0.87]

*Note:* All measures were POMP‐transformed. Estimates in bold do not include zero in their 95% CIs.

Abbreviations: Rel., between‐person reliability; SD, standard deviation; Stab., meta‐analyzed 1‐year stability based on the Spearman–Brown‐corrected correlation between the subsequent waves in Dataset 3 and Wave 1 and 3 in Dataset 5.

^a^ Given that we did not have access to the individual neuroticism items in every dataset, we took the Cronbach's α from prior research (Kalokerinos et al. 2020) and computed it for the added datasets and then reported the mean reliability here in terms of Cronbach's α.

^b^ Reliability was estimated using the Spearman–Brown‐corrected split‐half reliability on odd and even days (e.g., Wendt et al. 2020).

^**c**^ Estimates are significantly heterogenous (*I* ^2^ > 50% and *p*_*Q*_ < 0.05).

### 2.3 Measures

#### 2.3.1 Affect

In each dataset, affect was assessed by asking participants how they were currently feeling, followed by a list of emotions (see Table S1). These emotions were rated on sliders or Likert scales that used different anchors and ranges of values. To facilitate interpretation and comparison, we harmonized the different scales by calculating the Percent of Maximum Possible Score (POMP; Cohen et al. 1999) of each emotion item. To do this, we subtracted the minimum possible score of an emotion item from the actual score and divided this difference by the difference between the maximum and minimum of the measure. Finally, we multiplied it by 100, so that the POMP scores range from 0 to 100, with, for example, a 40 in anger indicating 40% of maximum anger. Importantly, this does not change the distribution itself, but transforms the range of values linearly. Finally, we calculated affect balance as a measure of affective well‐being (Park et al. 2023).

#### 2.3.2 Neuroticism

Several Big Five personality questionnaires were used to assess neuroticism across the individual studies (see Table S1). In six studies, participants completed the Big Five Inventory (BFI), which assesses neuroticism with eight items on a scale ranging from 1 (*disagree strongly*) to 5 or 7 (*agree strongly*), depending on the dataset. The neuroticism subscale showed a good reliability, $\alpha_{\text{mean}}$= 0.85 (range: 0.81–0.92). Five studies used the Ten‐Item Personality Inventory (TIPI; Gosling, Rentfrow, and Swann 2003), which assesses emotional stability with two items on a scale ranging from 1 (*disagree strongly*) to 7 (*agree strongly*). To obtain a measure of neuroticism, we reverse‐scored the emotional stability subscale, which yielded a mean reliability of *α* = 0.65 (Range: 0.55 to 0.72). Another two studies used the 12‐item version of the neuroticism subscale from the Revised NEO Personality Inventory (NEO‐PI‐R; Costa Jr. and McCrae 2008), using a scale ranging from 1 (*strongly disagree*) to 5 (*strongly agree*) and demonstrating good mean reliability of *α* = 0.84 (range: 0.83–0.85). Finally, one study used the 12‐item Negative Affectivity subscale of the Personality Inventory for the DSM‐5 (PID‐5; Krueger et al. 2012), which assesses neuroticism via 12 items on a 4‐point Likert scale ranging from 0 (*very false or often false*) to 3 (*very true or often true*). Again, we computed POMP scores for ease of interpretation and comparison.

### 2.4 Analytic Approach

All datasets were prepared in Stata 17 (College Station, TX, USA: StataCorp LP) and analyzed in Stata 17 and Mplus version 8.10 (Muthén & Muthén, 1998–2021). We conducted several sets of analyses on each dataset using dynamic structural equation modeling and then meta‐analyzed the results using a restricted maximum likelihood estimator on the Fisher's *z*‐transformed estimates. In the result section, we present the standardized coefficients (using Mplus' STDYX standardization option) along with their 95% credible intervals (95% CI) as well as heterogeneity statistics Cochran's *Q* and *I* ^2^. We viewed the meta‐analytically derived mean association as heterogenous if both Cochran's *Q* indicated significance (*p* < 0.05) and the *I* ^2^ statistic was at least 50% (Higgins and Thompson 2002). Recent guidelines (Funder and Ozer 2019) were used to interpret the size of the associations, with the following cut‐offs: β = 0.05 for very small, β = 0.10 for small, β = 0.20 for medium, β = 0.30 for large, and β = 0.40 for very large associations. To judge the significance of the results, we used an α = 0.05 and report 95% confidence and credible intervals.

For the estimation of the models in Mplus, we used two MCMC chains with a Bayesian estimator, the default Gibbs sampler, the default priors so that the results are driven by the data, a thinning of 20, and a convergence cut‐off by setting the Potential Scale Reduction to a recommended value of 1.005 (Zitzmann and Hecht 2019). A thinning of 20 was used to reduce autocorrelations between the iterations. Moreover, we required a total number of 2000 iterations before terminating model estimation if the convergence cut‐off was met. All models were checked for signs of misspecification, which we did not find: All autocorrelations were low (*r* < 0.20 or decaying rapidly) and the trace plots resembled the desired shape of a fat, hairy caterpillar.

#### 2.4.1 Reliability and Stability

To estimate the reliability of our measures, we computed the Spearman–Brown‐corrected split‐half reliability (Eisinga, te Grotenhuis, and Pelzer 2013) on odd and even days (e.g., Wendt et al. 2020). We then used these reliabilities to correct for the influence of measurement error in the main analyses testing our hypotheses (Parsons, Kruijt, and Fox 2019). We also examined the 1‐year stability of the study measures in the two samples that longitudinal data were available. Dataset 3 included four waves, each separated by 1 year. Dataset 5 included three waves, with the first and third waves separated by 1 year. We therefore calculated the Spearman–Brown‐corrected correlation between the respective waves, averaged the reliability coefficients from Dataset 3, and performed a meta‐analysis in Stata 17 on the two estimates per measure (one from Dataset 3 and one from Dataset 5).

#### 2.4.2 Hypothesis 1: Concurrent Relation Between iM and iSD

To test Hypothesis 1 that the *iM* is less strongly associated with the *iSD* for affect balance than for negative affect, we computed a Bayesian location scale model in Mplus 8.10 for the respective datasets, with observations nested within participants. In these models, the random intercepts of affect correspond to the affective level (i.e., *iM*). Unlike in standard multilevel models, the residual terms (i.e., any differences that could not be explained by the random intercept) were allowed to be different for each participant, which could then be used as a measure of individual differences in affective variability. These level‐1 random parameters then become latent level‐2 variables and are thus not treated as perfectly reliably assessed observed variables. This approach not only avoids underestimation of standard errors and inflated type I errors associated with a two‐step approach of computing the standard deviation in one model and using it in a second model (Liu, Kuppens, and Bringmann 2021) but also accounts for the different number of sampled occasions per participant (Lüdtke et al. 2008).

To test Hypothesis 1, we estimated the person‐specific variance of negative affect and affect balance and regressed each on mean negative affect or affect balance, respectively, in Mplus. In addition to including the linear mean as a predictor, we also included the squared *z*‐standardized measure of affect^6^ to examine the quadratic association with the affective variability (*iSD*) as described in the introduction.

#### 2.4.3 Hypothesis 2: Relationship Between Neuroticism and Variability in Affect Balance

To test Hypothesis 2, we again first extracted the person‐specific residual term as a measure of affect balance variability in Mplus and then used it at the between‐person level as an outcome of neuroticism, along with both mean and squared mean affect balance to control for linear and quadratic confounding. In addition, we predicted mean affect balance by neuroticism in the same model. We then repeated the analyses for negative affect and also computed models, in which neuroticism was the outcome and variability in affect balance, mean and squared mean affect balance the predictors. Finally, we corrected for the influence of measurement error and meta‐analyzed the results.

#### 2.4.4 Hypothesis 3: Current Affect Balance as a Moderator of the Relationship Between Neuroticism and Variability in Affect Balance

To test Hypothesis 3, in which we hypothesized that individuals high versus low in neuroticism would report greater fluctuations in affect balance for high levels of affect balance but smaller fluctuations for low levels of affect balance, we first computed the square root of the squared difference (*RSSD*) at each measurement occasion by subtracting the previous affect balance at *t* − 1 from the current affect balance at *t* (except for a participant's first measurement occasion), squaring the difference and then taking the square root in Stata 17. When aggregated at the person‐level, this measure is known as the individual root mean square of successive differences (*iRMSSD*; Jahng, Wood, and Trull 2008), which has been interpreted as affective instability in emotion research (e.g., Dejonckheere et al. 2019). We use this measure to capture momentary fluctuations in affect instead of the *iSD* because the *iSD* cannot be computed at the within‐person level. While the *iRMSSD* has the advantage over the *iSD* of accounting for the temporal order in the observations, it also has several disadvantages. Mainly, it is the product of the *iSD* and the autocorrelation (Mun et al. 2019), and by combining these measures into a single measure, the *iRMSSD* may obscure important relationships between the measures and neuroticism when the *iSD* and the autocorrelation are inversely associated with another variable. Thus, some researchers argue that it is better to report both the *iSD* and the autocorrelation if one is interested in the temporal characteristics of affect (Wang, Hamaker, and Bergeman 2012). Consequently, we present both the results for *iSD* and the *iRMSSD* and use the *RSSD* to examine the associations at the within‐person level and the moderation by neuroticism. To do this, we first ran a model in Mplus version 8.10, in which we first predicted the *iRMSSD* at the between‐person level by neuroticism, controlling for mean affect balance and squared mean affect balance, which was an additional test of Hypothesis 2 using the *iRMSSD* instead of the *iSD*. Next, we ran a model, in which momentary instability as assessed by the *RSSD* was predicted by prior affect balance at *t* − 1 at the within‐person level. We allowed for a random slope and regressed it, as well as the momentary instability and mean affect balance, on neuroticism at the between‐person level.

## 3 Results

### 3.1 Preliminary Analyses

Before testing our hypotheses, we conducted some preliminary analyses. First, we examined how strongly neuroticism was linked to negative affect but also to positive affect. As shown in Table 1, neuroticism was significantly associated with more negative affect in daily life, $\bar{\beta }$ = 0.40. It was also significantly associated with less positive affect, $\bar{\beta }$ = −0.35, with a mean effect size similar to that for negative affect. In addition, when entering mean negative affect first to a model predicting neuroticism and then entering mean positive affect, mean positive affect explained an additional 6.5% of the total variance in neuroticism above and beyond the 9.2% that mean negative affect explained in the first step. Thus, we can conclude that positive affect should not be neglected when examining the relationship between neuroticism and affect.

Affect balance, however, showed the largest association with neuroticism, $\bar{\beta }$ = 0.44. Moreover, when predicting neuroticism, positive and negative affect explained 15.8% of the total variance in neuroticism, while affect balance explained 15.4%, a marginal difference of 0.4%. Thus, these results demonstrate the utility of integrating positive and negative affect into a single measure of affective well‐being.

### 3.2 Hypothesis 1: Relation Between iM and iSD

**FIGURE 1:**
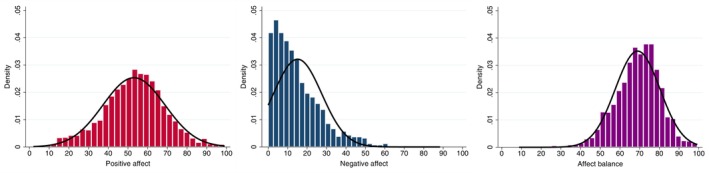
Distribution of mean positive affect (left panel), mean negative affect (middle panel), and affect balance (right panel).

In Hypothesis 1, we hypothesized that mean affect balance would be less strongly correlated with variability in affect balance than for negative affect because it is less right‐skewed. Figure 1 shows the distribution of positive affect, negative affect, and affect balance. As assumed, negative affect was much more right‐skewed than affect balance and positive affect.

**FIGURE 2:**
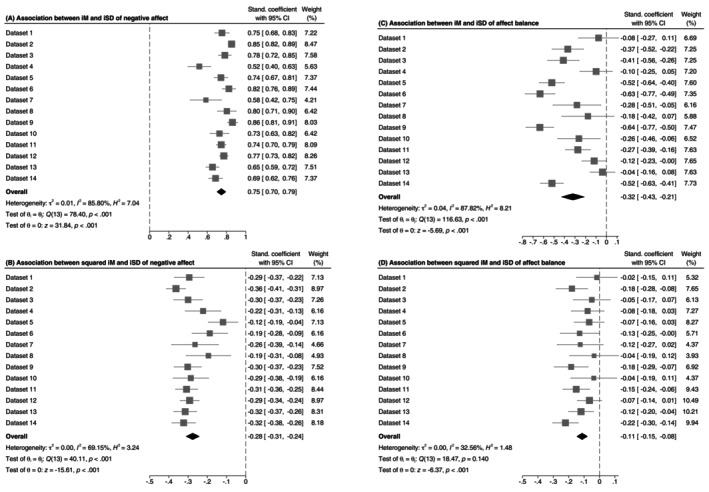
Meta‐analysis of the association (slope coefficients) between mean negative affect (A) or squared mean negative affect (B) and variability in negative affect, and between mean affect balance (C) or squared mean affective well‐being (D) and variability in affect balance.

To test Hypothesis 1, we predicted variability in affect balance or negative affect by both the mean and the squared mean of affect balance or negative affect to reflect both linear and quadratic associations. For negative affect, we found a very large mean linear association, $\bar{\beta }$ = 0.75. Although the test for heterogeneity was significant, the individual associations ranged from β = 0.52 to 0.86 and were significant and very large in all datasets (see Figure 2A). The mean quadratic association was also significant, $\bar{\beta }$ = −0.28, with the individual associations ranging from −0.36 to −0.12 (Figure 2B), indicating small to large associations in all datasets.

In turn, the mean linear mean‐variability association was much smaller for affect balance, $\bar{\beta }$ = −0.32.^7^ Although still significant and large in size, it was far from values indicative of multicollinearity problems (Dormann et al. 2013), which was not the case for negative affect. As illustrated in Figure 2C, the affect balance distributions followed a normal distribution in most datasets, with the location of the central point (i.e., the mean) shifted toward the positive end of the scale, ranging from *M* = 63.9 (Dataset 13) to *M* = 75.7 (Dataset 9). Although the mean quadratic association was also significant (Figure 2D), $\bar{\beta }$ = −0.11, it was only very small and did not exceed an absolute value of 0.23 in any of the datasets. Therefore, we found support for Hypothesis 1 that affective variability is less strongly correlated with the mean affect for affect balance than for negative affect.

### 3.3 Hypothesis 2: Relationship Between Neuroticism and iSD

**FIGURE 3:**
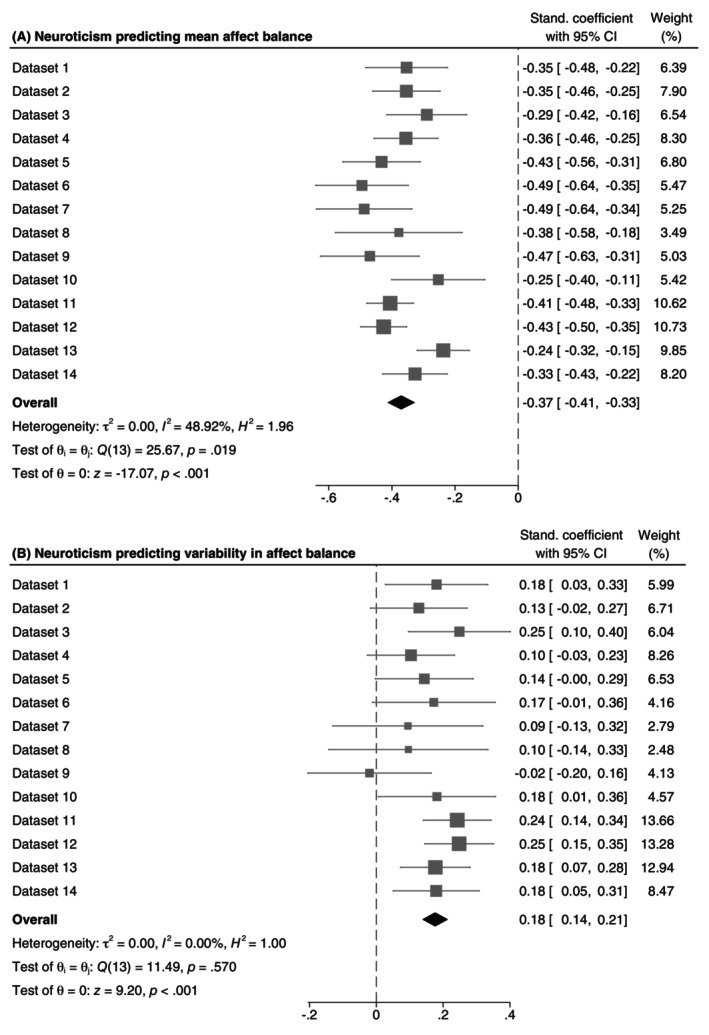
Forest plot of the association between neuroticism and mean affect balance (panel A) and affect balance variability (panel B), controlling for squared mean affect balance. Stand., standardized.

In Hypothesis 2, we predicted that neuroticism would positively be related to variability in affect balance over and above mean affect balance. To this end, we predicted mean affect balance and variability in affect balance by neuroticism, controlling for both mean and squared mean affect balance when predicting variability in affect balance. As shown in Figure 3A, neuroticism was significantly associated with lower mean affect balance in daily life, $\bar{\beta }$ = −0.37. Importantly, neuroticism was also significantly associated with increased variability in affect balance, both when controlling for mean affect balance, $\bar{\beta }$ = 0.18 (Figure 3B), and when not, $\bar{\beta }$ = 0.28 (Table 1). Given that this mean association was medium in size and not significantly heterogeneous and given that the *iSD* of affect balance showed good reliability and stability, we conclude that we found support for Hypothesis 2, such that participants high compared to low in neuroticism did indeed report greater variability in affect balance above and beyond mean affect balance.

In addition, we conducted two supplementary analyses. First, we predicted neuroticism from the *iSD, iM*, and *iM* ^2^ of affect balance. As shown in the online supplement (Figure S3 in the online supplement at https://osf.io/wtasq?view_only=d97ddf7e9d29430f9c951a1daccca02f), variability in affect balance significantly predicted neuroticism over and above the *iM and iM* ^2^ of affect balance, $\bar{\beta }$ = 0.23, 95% [0.18, 0.28], *I* ^2^ = 0.01%, *p*_*Q*_ = 0.721. Second, one might argue that much information about affective variability is lost by using affect balance rather than examining positive and negative affect separately. For example, experiencing a positive affect score of 70 and a negative affect score of 60 would result in the same affect balance score of 10 as experiencing a positive affect score of 20 and a negative affect score of 10. Thus, the affect balance would be stable and not indicative of variability, even though both positive and negative affect fluctuate. However, this is unlikely for several reasons. First, positive and negative have a very large, negative mean association (see Table 1), so fluctuations of positive and negative affect in the same direction do not occur often. Second, as shown in Table 1, variability in positive affect and variability in negative affect were highly correlated, $\bar{\beta }$ = 0.59. Expressing these mean associations in terms of internal consistency yielded an acceptable Spearman–Brown‐corrected reliability (Eisinga, te Grotenhuis, and Pelzer 2013) of $r_{\mathrm{SB}}=\frac{2\times .58}{\left(1+.58\right)}$ = 0.73, providing evidence of internal consistency for using them as a single construct. Finally, when neuroticism was predicted by mean affect balance and variability in affect balance, the two predictors explained 17.4% of the variance in neuroticism, compared with 17.5% when mean positive affect, mean negative affect, and variability in positive and negative affect were included to explain variance in neuroticism. Thus, we found no evidence of information loss when using affect balance. However, we predicted variability in negative affect from neuroticism and controlled for the *iM* and *iM* ^2^ of negative affect (Figure S4 in the online supplement). We found a mean association of $\bar{\beta }$ = 0.07, 95% [0.04, 0.10], *I* ^2^ = 18.6%, *p*_*Q*_ = 0.376, which is in line with mean association between neuroticism and the *iRSD* of negative affect reported by Kalokerinos et al. (2020). We also predicted variability in positive affect (Figure S3) and found a mean association of $\bar{\beta }$ = 0.12 95% [0.08, 0.15], *I* ^2^ = 0.1%, *p*_*Q*_ = 0.799. Given that the two mean associations were smaller than that for affect balance, this further supports the utility of using an integrated affective well‐being measure such as affect balance.

### 3.4 Hypothesis 3: Current Affect Balance as a Moderator of the Relationship Between Neuroticism and Variability in Affect Balance

Finally, we tested the notion that neuroticism is differently associated with instability in affect balance, depending on current affect balance levels. Before testing Hypothesis 3, we wanted to test whether the results regarding the relationship between neuroticism and variability in affect balance were the same when using the momentary instability measure aggregated at the between‐person level, the *iRMSSD*, instead of the *iSD*. The results showed that, on average, neuroticism was significantly associated with increased instability at the between‐person level, $\bar{\beta }$ = 0.17, 95% [0.09, 0.25], *I* ^2^ = 68.2%, *p*_*Q*_ < 0.001 (see the forest plot in Figure S5 in the online supplementary material for more information and see Figures S6 and S7 for the results for positive and negative affect). Given that the mean association was the very close to the one when using the *iSD* (see Figure 3), the results support the suitability of our momentary instability measure for examining within‐person associations at the within‐person level.

**FIGURE 4:**
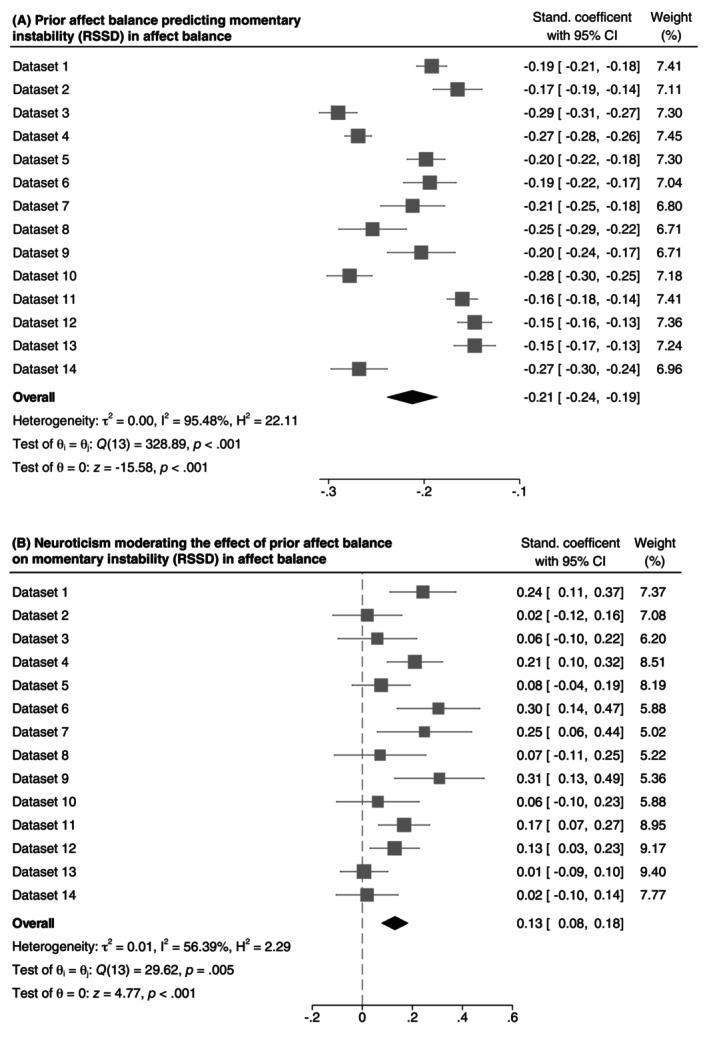
Forest plot of (A) the effect of prior affect balance on momentary instability and (B) its moderation by neuroticism.

**FIGURE 5:**
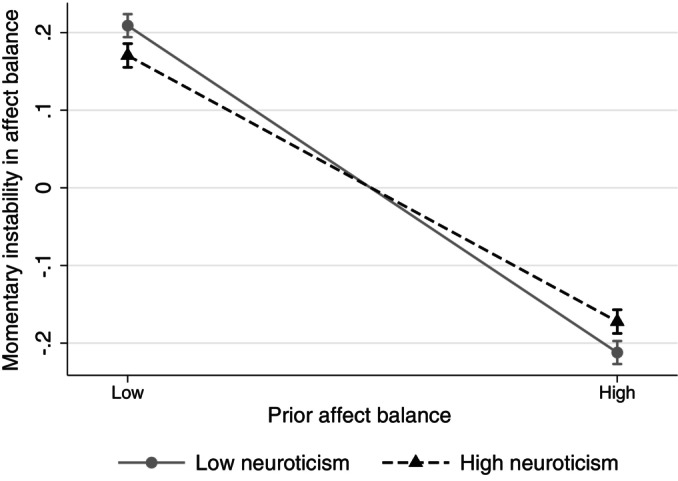
Cross‐level interaction of neuroticism and prior affect balance in momentary instability in affect balance.

Next, we tested Hypothesis 3. First, we found that current affect balance was significantly associated with weaker subsequent fluctuations in terms of lower momentary instability, $\bar{\beta }$ = −0.21 (see Figure 4). This indicates that when a participant felt better than usual, their affect balance was, on average, more stable than when they felt worse than usual. Importantly, neuroticism was significantly associated with the within‐person association between current affect balance and subsequent fluctuations in affect balance, $\bar{\beta }$ = 0.13, 95% [0.08, 0.18], *I* ^2^ = 56.4%, *p*_*Q*_ = 0.005. As illustrated in Figure 5, when affect balance levels were lower than usual (person mean minus one standard deviation), participants high in neuroticism reported significantly weaker subsequent fluctuations in affect balance than participants low in neuroticism, *b* = −0.04, 95% CI [−0.07, −0.01]. Conversely, when affect balance levels were higher than usual (person mean plus one standard deviation), participants high in neuroticism reported significantly stronger subsequent fluctuations in affect balance than participants low in neuroticism, *b* = 0.04, 95% CI [0.01, 0.07]. Thus, we found support for Hypothesis 3, that neuroticism is associated with stronger variability instability for better affective well‐being, but weaker instability for worse affective well‐being.

## 4 Discussion

Emotional variability is a fundamental feature of neuroticism. However, recent research has suggested that the relationship between neuroticism and daily affective fluctuations may be misleading because of confounding between the *iM* and *iSD* of negative affect (Kalokerinos et al. 2020). In the present study, we sought to illustrate that this spurious association may be the result of the non‐normal distribution of negative affect. Indeed, when we reanalyzed 14 ambulatory assessment datasets and controlled for the *iM* and the *iM* ^2^, we found that the concurrent relationship between neuroticism and variability in negative affect was significant but very small, $\bar{\beta }$ = 0.07 (Spearman–Brown corrected). However, when we used affect balance as a measure of affective well‐being, which is much more normally distributed and has fewer extreme values than negative affect, we found a medium‐sized mean association between the two, $\bar{\beta }$ = 0.18 (Spearman–Brown corrected). Thus, by using an affect scale that is less strongly affected by the confound between the *iM* and the *iSD*, we find clear evidence for the notion of increased affective variability in individuals high vs. low in neuroticism. This finding demonstrates that ignoring the violations of the normality assumption may also lead to very different conclusions.

Our finding is consistent with research showing that neuroticism was significantly associated with variability in negative affect when a Bayesian censored location‐scale model was used to model and predict variability in negative affect (Mader et al. 2023). A censored model can model out‐of‐scale values, such as a value less than 0 on a negative affect scale ranging from 0 to 100, thereby recreating the unbounded normal distribution that negative affect is thought to follow. It should be noted, however, that applying a censored model to out‐of‐scale values is not without its problems. While a censored model can be used to statistically model out‐of‐scale values, it is important to consider whether these values can actually exist. For example, if you were to use a 2‐m tape measure to determine a person's height, you would only know that a person taller than two meters is at least two meters tall. However, this is not the case with many negative affect scales, where the lower endpoint of negative emotions is often labeled “not at all” when asked if a participant is currently experiencing an emotion.

However, one disadvantage of the affect balance measure might be that information might be lost compared to using positive and negative affect separately and that potentially different associations for positive and negative affect might be obscured. Importantly, we did not find evidence for these potential issues in this line of research. Not only did affect balance explained the same variance in neuroticism as both positive and negative affect, variability in positive affect and in negative affect was highly correlated, as evidence by a reliability of 0.73 that provides evidence for using them as a single construct, for example, by using variability in affect balance. Moreover, variability in positive affect was also similarly associated with neuroticism as variability in negative affect. Thus, we believe that using affect balance as a measure of affective well‐being is a simple way to avoid the potential confounding issues when modeling both the mean and variability of affect.

However, we also linked to research on early warning signals, which views affective variability differently depending on the current state of the affective system. To date, research on affective variability and neuroticism has assumed that variability reflects some form of instability and that this reflects difficulties in maintaining high levels of affective well‐being. However, when affective well‐being is at low levels, variability would indicate change and thus be adaptive for an individual. We tested this hypothesis and found evidence that current levels of affect balance are a moderator of the association between neuroticism and variability. Specifically, participants high compared to low in neuroticism found it more difficult to maintain high levels of affective well‐being, as reflected in stronger subsequent fluctuations in affect balance when currently feeling better than usual, but also more difficult to improve low levels of affective well‐being, as reflected in weaker subsequent fluctuations in affect balance when currently feeling worse than usual. This may explain why previous research has found that neuroticism is associated with both more variable and more persistent affect, resolving the stability–instability paradox. Thus, increased variability should not be seen as an unequivocally bad sign, but rather as a sign that an affective system is changing, which may be adaptive or maladaptive for an individual, depending on the initial state of the affective system. This finding fits well with a recent study that found that variability in negative affect, but not in positive affect, was differentially associated with depressive symptoms, depending on the person's mean negative affect (Maciejewski et al. 2023). Thus, future research on affective variability should consider the context in which it is examined and use this information to develop interventions for neuroticism. These interventions could aim to stabilize the affective system during good phases of life and incentivize change during worse phases.

### 4.1 Limitations and Future Research Directions

In the present research, we used the *iSD* (or, rather, the person‐specific residual term of affect balance) to capture affective variability. One major disadvantage of the *iSD* is that it is insensitive to the temporal order of the observations and thus cannot capture how frequent affect fluctuates. Thus, two participants can have the same *iSD* (say 2 for a scale ranging from 0 to 4), although their experience might differ: One person might experience no negative affect for the first 3 days and then stronger negative affect for the next 3 days (i.e., 0, 0, 0, 3, 4, 4), while another person might experience strong fluctuations (i.e., 0, 3, 0, 3, 0, 4). In this example, the affect of the first person is more stable than the affect of the second person, who experiences more fluctuations. Thus, the best way to think about the *iSD* is that it is an assessment of the extremes of values for a given mean, that is, the amplitude of fluctuations from mean affect balance in daily life. For example, if the average affect balance on a scale of 0–100 is 50, a large *iSD* indicates that a person experiences very high highs and very high lows in their daily life, whereas a very small *iSD* indicates a stable affect balance centered around 50, with few very high highs and few very high lows. However, accounting for the temporal order by using the *iRMSSD* showed a very similar mean association between neuroticism and instability in affect balance, which further supports Hypothesis 2.

Another drawback of the *iSD* is that it is not clear how the data is actually generated. In this study, we chose a descriptive research perspective and thus did not seek to define and test underlying affective processes or mechanisms that may explain why neuroticism is significantly associated with better mean affect balance and greater variability in affect balance in daily life. However, we focused on affective variability because previous research (Kalokerinos et al. 2020) has challenged the basic notion of an association between neuroticism and affective variability, and thus it is important to first establish this relationship before attempting to explain it. Furthermore, recent research has shown that advanced affect dynamic measures such as affective inertia (i.e., the autocorrelation between successive measurement occasions of affect), do not explain sufficient variance in well‐being measures above and beyond mean affect and affective variability (Dejonckheere et al. 2019). Thus, future research could use generative modeling (Pulcu et al. 2022) that describe the underlying affective processes that cause an outcome or by finding ways to capture affective processes in daily life that map more closely onto the data‐generating mechanisms, such as affective reactivity or recovery (e.g., Suls and Martin 2005), that are less affected by conceptual issues (Dejonckheere et al. 2019).

Finally, although affect balance has several advantages in terms of a more favorable distribution regarding the confounding of the *iSD* with the *iM* and in terms of including both negative and positive affect in one measure of affect, it is not without disadvantages. Mainly, some information may be lost when using affect balance. For example, if positive and negative affect were high (e.g., 5 on a scale of 0–6) on one measurement occasion then low (e.g., 1) on the next, this change would not be reflected in affect balance. While this is a potential problem, we do not believe that it confounded the present research. First, PA and NA were highly negatively correlated within‐person, so it is not often the case that both change in the same direction (e.g., from high to low). In addition, in only 2.6% of all observations did PA and NA change in the same direction with a substantial difference, i.e., a change of 10 scale points (i.e., a 10% change in scale). Second, variability and instability in both positive and negative affect were highly correlated with variability and instability in affect balance, with all mean associations greater than 0.85. Thus, in most cases, a change in positive or negative affect meant a change in affect balance. Third, affect balance is based on the notion that PA outweighs the effects of NA, and thus a change in PA from 5 to 1 and in NA from 5 to 1 on a scale of 0 to 6 might still indicate to a person that his or her affective well‐being is only moderate. Whether this is actually the case cannot be answered with the datasets we had access to but would require new data.

### 4.2 Conclusion

Our meta‐analysis illustrates the importance of measurement and modeling when assessing associations between neuroticism and affective variability. By using affect balance as a measure of affective well‐being, which is less affected by the *iM*‐*iSD* confound, we found a significant and medium‐sized association with neuroticism. Based on our findings, we recommend that future research focus on understanding the affective processes and mechanisms behind why individuals high in neuroticism (as opposed to those low in neuroticism) report poorer affect balance and greater affect variability. This will require addressing the conceptual and empirical challenges of studying these processes in everyday life.

## Author Contributions

**Mario Wenzel**: conceptualization, methodology, formal analysis, investigation, data curation, writing–original draft. **Whitney R. Ringwald**: investigation, writing–review & editing. **Aleksandra Kaurin**: investigation, writing–review & editing. **Oliver Tüscher**: investigation. **Thomas Kubiak**: investigation. **Aidan G. C. Wright**: investigation, writing–review & editing, funding acquisition.

## Conflicts of Interest

The authors declare no conflicts of interest.

## Supporting information


Data S1.

## Data Availability

All data are available on OSF (https://osf.io/t56a4/?view_only=d97ddf7e9d29430f9c951a1daccca02f) and the data we included from Kalokerinos et al. (2020) can be found at https://osf.io/gvfdx/.

## Endnotes

^1^
While many different variability values are possible in the middle of a scale, there are fewer possible values closer to the boundaries. Thus, as the means approach the extremes of a scale, the total possible variability becomes constrained and approaches 0. In other words, individuals with very low or very high *iM* levels cannot experience large deviations from their mean affect levels, or else they would not have very low or very high *iM* levels, making their affect less variable. For example, consider an affect scale ranging from 0 to 10 and three measurement occasions. An *iM* = 5 could be derived from a distribution with no variability (5, 5, 5), minimum variability of *iSD* = 1 (4, 5, 6), or maximum variability of *iSD* = 5 (0, 5, 10). However, to obtain an *iM* = 0.33, there is a more limited range of variability—the maximum possible *iSD* = 0.50 (0, 0, 1). And, empirically, the magnitude of variability values near the bounds is limited and often smaller than values near the center of the scale.

^2^
There are alternative ways to deal with the confound between *iM* as a measure of affective baseline and the *iSD* as a measure of affective variability. One way to solve this issue is to use the iMode. Using such approach, it was found that the *iSD* showed a small mean association of $\bar{r}$ = 0.16 across 15 ambulatory assessment datasets when controlling for the *iM* and of $\bar{\beta }$ = 0.26 when controlling for the mode (Ringwald and Wright 2022). However, it is unclear how the iMode relates to affective variability when it is not captured by the *iSD*, which is based on the *iM*, but on a fitting dispersion measure such as Shannon's entropy (Shannon 1948). Another way would be to transform the negative affect scale to reduce the right‐skewness, for example, by applying a log transformation (Wenzel and Kubiak 2020). However, this would not solve the mismatch between the data‐generating process, which includes many observations near the minimum bound of negative affect, and the model. Moreover, applying a log‐transformation to negative affect would not only increase the likelihood of obtaining normalized residuals but would also change the ratio scale properties of this scale (Lo and Andrews 2015): For example, a difference between a negative affect value of 20 and 10 on a scale ranging from 0 to 100 would yield the same log value (log(20) – log(10) = 0.30) than a difference between 60 and 30 (log(60) – log(30) = 0.30), although the latter difference (60–30 = 30) is three times as large as the first (20–10 = 10). Finally, a recent study used a Bayesian censored location scale model to model negative affect as a normal distribution censored by the affect scale, with values at 0 indicating values at or below the lower bound (Mader et al. 2023).

^3^
We would also like to emphasize that pitting *iSD* against *iM* is problematic from a theoretical point of view, since a strong association between these measures does not necessarily mean that variability is not an affective process underlying neuroticism. A detailed explanation can be found in the supplementary material on the OSF project page.

^4^
Please note that we did not include four studies from the meta‐analysis by Kalokerinos et al. (2020) given that three datasets were not collected using experience sampling but using a daily diary design (datasets 1–3 there). A fourth dataset did not provide the individual emotion items (dataset 11 there).

^5^
The number of observations is based on completed affect balance values.

^6^
To obtain this measure, we first averaged affect at the person level, *z*‐standardized these person means, then squared them (all in Stata 17), and finally included them as a between‐person (level‐2) variable in Mplus.

^7^
Please note that the negative sign of the mean‐variability association for affect balance does not indicate a reversed association compared to negative affect. Instead, affect balance is computed by the difference between positive minus negative affect and, thus, can be interpreted as an affective well‐being measure, which is inversely associated to negative affect. Thus, affect balance is bounded by the right bound, whereas negative affect is bounded by the left bound in the data. The difference can be illustrated by the assumed quadratic association (U‐Shape): If the scale is split in the center, there would be a positive linear association on the left side and a negative linear association on the right side.
