# Supplementary material for: Neuroticism is Associated With Greater Affective Variability at High Levels of Momentary Affective Well‐Being, but With Lower Affective Variability at Low Levels of Momentary Affective Well‐Being
Source: J Pers. 2024 Sep 6;93(3):740–54. doi: 10.1111/jopy.12972 (PMC12053825; doi:10.1111/jopy.12972)
Supplement: Supplementary file 1 — Data S1. [file JOPY-93-740-s001.pdf]

Supplementary Material

Relation between neuroticism and variability in negative affect as captured via the *iRSD* 2
Conceptual relation between mean affect and variability in affect..4
Table S1 7
Figure S1 9
Figure S2 10
Figure S3 11
Figure S4 12
Figure S5 13
Figure S6 14
Figure S7 15
References 16

Relation between neuroticism and variability in negative affect as captured via the *iRSD*

To control for this confound, Mestdagh et al. (2018) proposed the relative *iSD* (*iRSD*), which is computed by dividing a given *iSD* by the maximum *iSD* at a given *iM,* resulting in a possible range of 0 to 1. Using this *iRSD* instead of the *iSD*, Kalokerinos et al. (2020) found that the mean association between neuroticism and negative affect variability decreased from $\bar{r}$ = .28 to a non-significant $\bar{r}$ = .05. These results suggest that the relationship between neuroticism and negative affect variability may be the result of confounding with the *iM*.

Although the meta-analytic correlation suggests that the *iM* and *iRSD* are not significantly associated, $\bar{r}$ = -.06, 95% CI [-.22, .10] (Wenzel & Kubiak, 2020), the individual associations within the datasets showed a large and significant heterogeneity (I^2 = 88.7%). While five datasets showed a non-significant association below |.10|, one dataset showed a significant and very large positive association, and four datasets showed significant and large negative associations around *r* = -.35. Thus, in about half of the datasets, the *iM~iSD* confound was not removed by the use of the *iRSD*, and in about one third it was reversed. Although this may only indicate that the *iRSD* removes error variance, the results suggest a large proportion of variability in the individual associations and thus coefficients are less reliable when using the *iRSD*. Importantly, when comparing Figure 2C in Kalokerinos et al. (2020) for the Neuroticism~*iRSD* relationship and Figure 1A in Wenzel & Kubiak (2020) for the *iM~iRSD* relationship, it is evident that the distributions of the individual effect sizes look very similar, i.e., the distribution of the effect sizes of the individual datasets in Figure 2C is almost mirrored in Figure 1A in Wenzel & Kubiak (2020). This relationship is illustrated in Figure 2 in the present research, where we show the z-standardized Neuroticism~*iRSD* relationship from Kalokerinos et al. (2020) and the z-standardized *iM~iRSD* relationship from Wenzel & Kubiak (2020). The results in Figure S1 suggest that differences in the Neuroticism~*iSD* associations could be explained by differences in the *iM~iRSD* associations. And indeed, not only did differences in the *iM~iRSD* associations fully explain

differences in the Neuroticism~*iRSD* relationship (Wenzel & Kubiak, 2020), but they were also highly correlated with variability in the Neuroticism~*iRSD* relationship, $r = .86$ (Wenzel et al., 2023). Importantly, this was not the case when *iSD* was used instead of *iRSD*. Thus, the transformation of the *iSD* into the form of the *iRSD* produced statistical artifacts in the form of an inverse confound that was highly related to the relationship of interest, the relationship between neuroticism and affective variability.

Furthermore, it has been demonstrated that the *iRSD* was not significantly associated with the number of negative affect episodes, $\bar{r} = .03$, another measure of negative affect variability, whereas the *iSD* showed a very large association, $\bar{r} = .67$ (Wenzel et al., 2023). Thus, only the *iSD* but not the *iRSD* showed convergent validity.

In addition, we argue in this paper that there is also a conceptual problem with the *iRSD*. The *iRSD* tries to solve the *iM~iSD* confound by standardizing the *iSD* to the maximum *iSD* for a given *iM*. This means that very small *iSDs* at the limits of the scale can get very large *iRSDs*. For example, consider the time series example from earlier, with a time series (0, 5, 10) with an *iM* of 5 and an *iSD* of 5, and another time series (0, 0, 1) with an *iM* of 0.33 and an *iSD* of 0.50. Calculating the *iRSD* would yield a value of 1 in both cases, indicating the maximum possible variability. However, it is very likely that the experiences are very different: Whereas the first time series reflects an individual who experiences very different intensities of negative affect, the second time series reflects an individual who experiences very little intensity or variability in their negative affect. Importantly, Mestdagh et al. (2018) did not provide a theoretical reason beyond statistical confounding for the decision to give stronger weights to values closer to the bounds. However, a characteristic of high or low levels of negative affect is that they do not fluctuate and the *iRSD* distorts this by representing minimal deviations in this range as extreme variability.

Finally, the skewed distribution of negative affect is a problem not only for the *iSD* but

also for the *iM*, since the calculation of the mean depends on symmetric distributions (e.g., normal), making it sensitive to outliers. More importantly, the mean can only be meaningfully interpreted as a measure of affective level if the scales are normally distributed. For example, Figure 3 shows the distribution of negative affect for an exemplar participant and illustrates that the mean does not represent the central tendency of the distribution. Ringwald and Wright (2022) recently argued that the median or mode of negative affect may be a better measure of central tendency because these indices are less sensitive to skew. As can be seen in Figure 3, the median and especially the mode are better representations of the center of the distribution for this participant.

Conceptual relation between mean affect and variability in affect

We would also like to emphasize that pitting *iSD* against *iM* is problematic from a theoretical point of view, since a strong association between these measures does not necessarily mean that variability is not an affective process underlying neuroticism. As proposed by Mõttus et al. (2020), personality research can be divided into three perspectives: descriptive, predictive, and explanatory, which differ in terms of their goals and the methods they use to achieve these goals. The descriptive research perspective focuses on the links between personality traits and their correlates, such as affective experiences, for example, by testing evolving models of how personality manifests (e.g., Wrzus & Roberts, 2017). In terms of the present research, the prototypical question would be: If neuroticism is conceptualized as people experiencing a poorer and a more variable affective well-being, is this actually the case in their everyday lives, i.e., can they be described in this way? Predictive personality research is interested in how well personality traits can predict outcomes. In addition, predictive research would include attempts to predict whether a person is likely to score high or low on neuroticism. In terms of the question of the present research, then, the prototypical question would be: Who is neurotic? Finally, the goal of explanatory research on neuroticism

and affect is not only to describe their relationship, but also to try to explain it by finding the causes of more reduced and variable negative affect in high vs. low neuroticism individuals, so the question would be: Why do individuals high vs. low in neuroticism report lower and more variable affective well-being?

Importantly, these different goals then lead to different approaches to examine and model the relationship between neuroticism and affective variability. To answer the descriptive question, one is interested in neuroticism as a predictor of the *iM* and *iSD* of affect balance (N → *iM* / *iSD*). One can, but need not, control for *iM* when examining *iSD* because both reflect important information, and their association provides meaningful information. For example, an individual who rarely experiences negative affect will have a very low mean and very low variability describing their affective experience. This is true for many other relationships, even on scales that are not bounded. For example, the temperature in countries closer to the poles is both lower and less variable on average than in countries between the poles and the equator. If you are planning a trip to the North Pole, both aspects are important, as you will need clothing to keep you warm, but not clothing for higher temperatures. The same is true for affective experiences, where the confound between the *iM* and the *iSD* at the bounds of the scale provides meaningful information. While a very high *iM* of affect balance indicates that one's affective well-being is very good, it is also very stable and thus reliable for a person. In other words, one may not need the *iSD* to predict whether an individual is highly neurotic if one already knows that the *iM* is very low or very high, but a very low variability still describes the affective experience of an *iM* at the bounds of a scale, which is highly relevant to the individual itself. In turn, in the middle of a scale, the confound is zero and, thus, many different *iSD* can be observed. Consequently, the confound between the *iM* and the *iSD* is not a statistical artifact, but rather reflects the properties of the data

generation mechanism (e.g., affective reactivity) and thus provides meaningful information if one is interested in descriptive personality research.

To answer the predictive question, one would model neuroticism as the outcome of mean affect balance and affective variability (*iM iSD* → N). Here, the confound is less important (apart from problems of multicollinearity) and does not provide meaningful information at the boundaries, since the reduced variability at the boundaries can already explain the lower or higher level of affect balance. Finally, to answer the explanatory question, one is interested in explaining the relationship between neuroticism and mean affect balance and variability in affect balance by examining potential mediators such as affective reactivity (e.g., Wrzus et al., 2021) or emotion regulation (e.g., Yang et al., 2020) (N → AR → *iM* / *iSD*).

In the present research, we were interested in the descriptive research question and thus considered neuroticism as a predictor of the *iM* and *iSD* of affect balance. We present the results of the analyses of affective variability, uncontrolled and controlled for mean affect balance, in the main text and the results of the analysis in which neuroticism is predicted by the *iM* and *iSD* in the online supplement.

Tables

Table S1

Sample characteristics

Data-set	*N*	Age in years: *M* (*SD*)	%female	Obs.	Days	S./day	Adher.	Emotions	Neurot.	Reference
1	125	22.9 (5.1)	77%	22,845	40	6	76.2%	PA: excited, happy, relaxed, satisfied; NA: afraid, angry, anxious, depressed, sad	BFI	Rowland et al., 2016
2	175	25.0 (5.4)	52%	10,095	7	12	68.7%	PA: excited, happy, relaxed, satisfied; NA: afraid, angry, anxious, depressed, sad	BFI	Wenzel et al., 2021
3	128	30.4 (9.2)	57%	4,907	7	6	91.1%	PA: excited, happy, relaxed, satisfied; NA: angry, anxious, depressed, sad	NEO-PI-R	-
4	176	27.2 (9.0)	67%	29,308	21	9-10	79.3%	PA: confident, happy, relaxed; NA: angry, sad, stressed	BFI	Grommisch et al., 2020
5	200	18.3 (1.0)	55%	12,293	7	10	87.9%	PA: excited, happy, relaxed; NA: angry, anxious, depressed, sad, stressed	TIPI	Erbas et al., 2018
6	96	19.1 (1.3)	63%	5,761	7	10	85.7%	PA: happy, relaxed; NA: angry, anxious, depressed, sad, stressed	TIPI	Brans et al., 2013
7	75	22.2 (5.3)	100%	4,520	10	7	86.1%	PA: confident, happy, relaxed; NA: angry, anxious, guilty, sad	BFI	Holland et al., 2017
8	78	23.7 (4.3)	100%	4,637	14	5	84.9%	PA: confident, happy; NA: afraid, angry, anxious, ashamed, embarrassed, guilty, sad	TIPI	Koval et al., 2019
9	85	26.5 (6.1)	100%	4.865	14	5	81.7%	PA: confident happy; NA: afraid, angry, anxious, ashamed, embarrassed, guilty, sad	TIPI	Koval et al., 2019
10	99	24.1 (6.9)	78%	8,618	14	7	88.9%	PA: happy, relaxed; NA: angry, sad, stressed	BFI	Dejonckheere et al., 2019
11	285	28.0 (6.4)	53%	20,151	14	6	84.2%	PA: confident, content, excited, happy; NA: angry, ashamed, guilty, nervous, sad, frustrated	NEO-PI-R 3	Vize et al., 2022
12	308	27.9 (5.0)	52%	17,454	10	7	81.0%	PA: confident, excited, happy, proud, relaxed; NA: angry, ashamed, hostile, nervous, sad	BFI	Ringwald et al., 2022
13	308	18.6 (1.0)	60%	11,463	10	5	74.4%	PA: confident, excited, happy, proud, relaxed; NA: angry, ashamed, hostile, nervous, sad	PID-5	Ringwald et al., 2022
14	250	19.8 (2.2)	76%	6,250	7	5	71.4%	PA: balanced, cheerful, happy, lively, relaxed; NA: afraid, angry, exhausted, sad, worried	TIPI	(Sosin et al., 2022)

Note. N = number of participants; %female = percentage of participants who identified themselves as females (compared to as males); Obs. = total number of observations (this is based on affect balance values and can, thus, differ slightly from previously reported adherence rates ; S./day = ambulatory signals per day; Adher. = adherence based on the participants included in the analyses Neurot. = Neuroticism; PA = positive affect; NA = negative affect; BFI = Big Five Inventory; TIPI = Ten-Item Personality Inventory; NEO-PI-R = Revised NEO Personality Inventory; PID-5 Personality Inventory for the DSM-5.

Figure S1

The z-standardized relationship between neuroticism and the iSD (from Figure 2C in Kalokerinos et al., 2020) and the z-standardized relationship between the iM and the iSD (from Figure 1A in Wenzel & Kubiak, 2020)

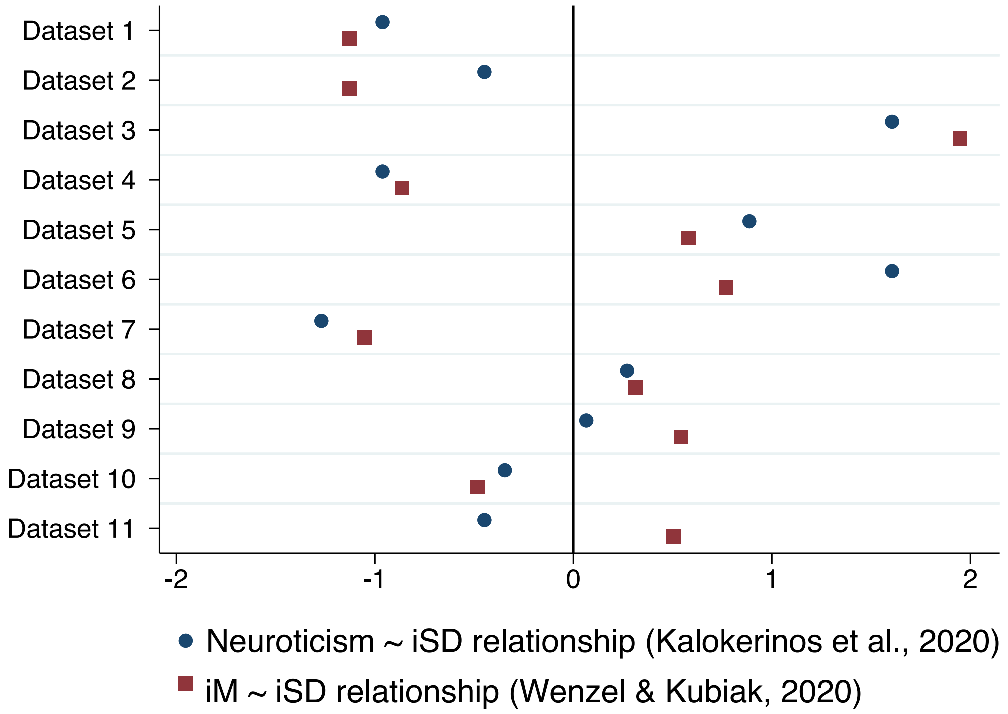

Note. iSD = intraindividual standard deviation of negative affect; *iM* = intraindividual mean of negative affect.

Figure S2

Histogram of Negative Affect of an Exemplar Participant in Dataset 1

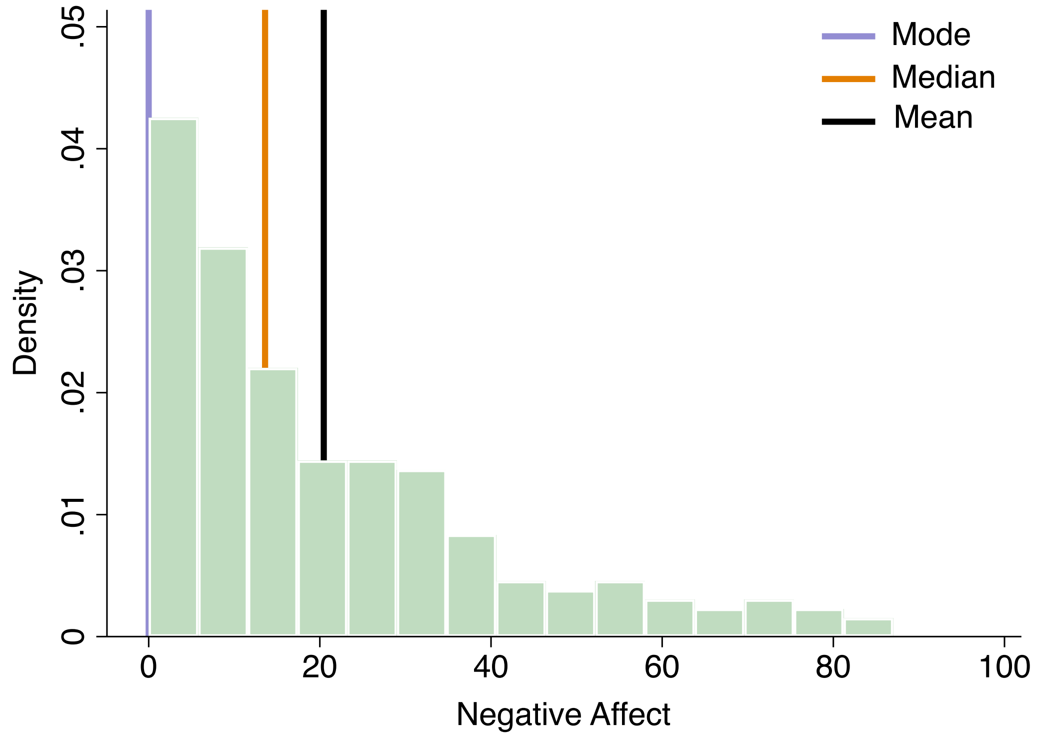

Figure S3

Forest plot of variability in affect balance predicting neuroticism

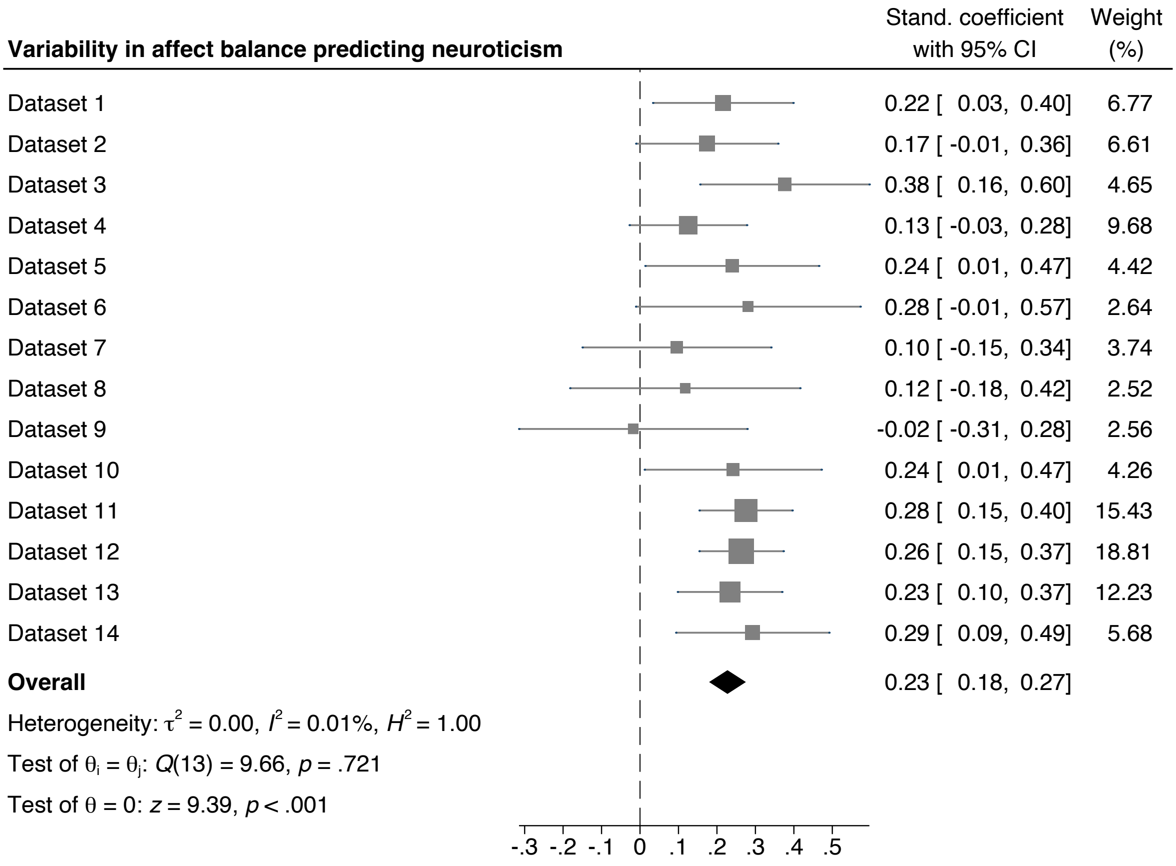

Note. Stand. = standardized.

Figure S4

Forest plot of neuroticism predicting variability in negative affect

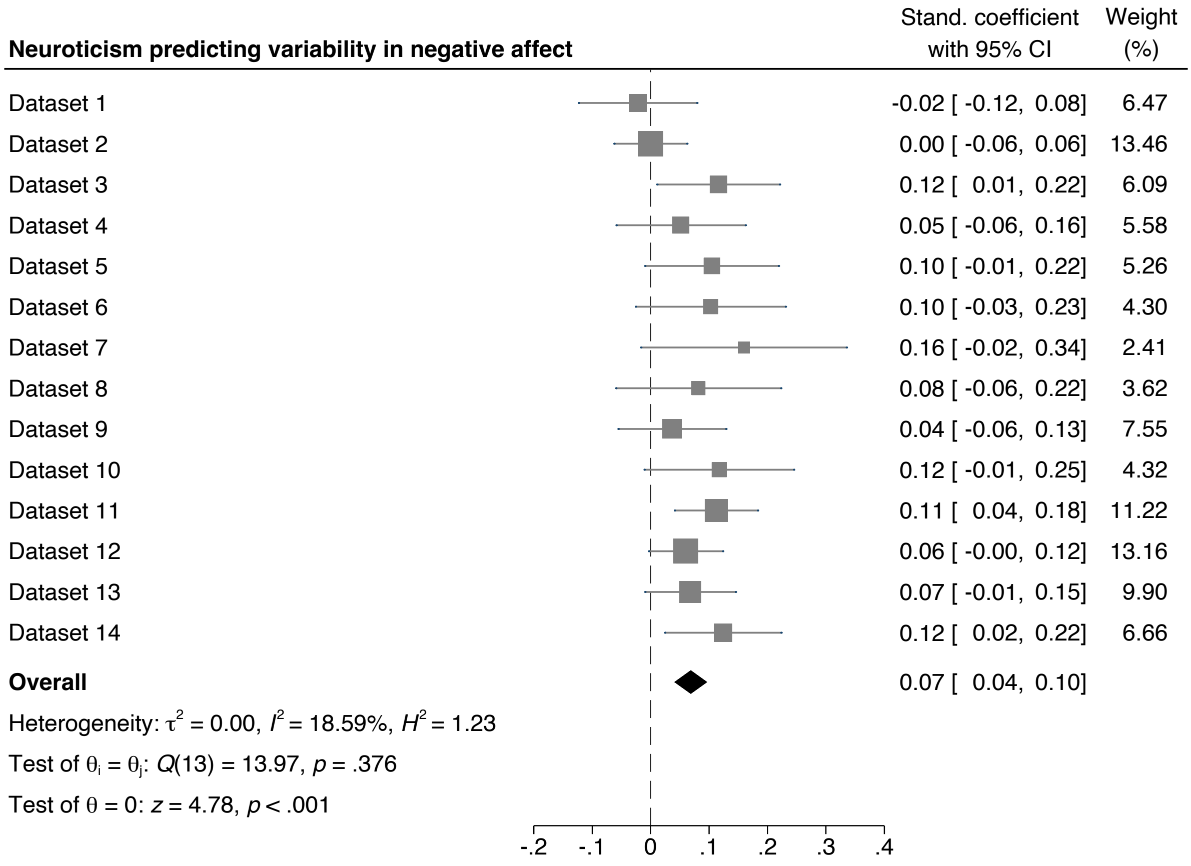

Note. Stand. = standardized.

Figure S5

Forest plot of neuroticism predicting instability in affect balance

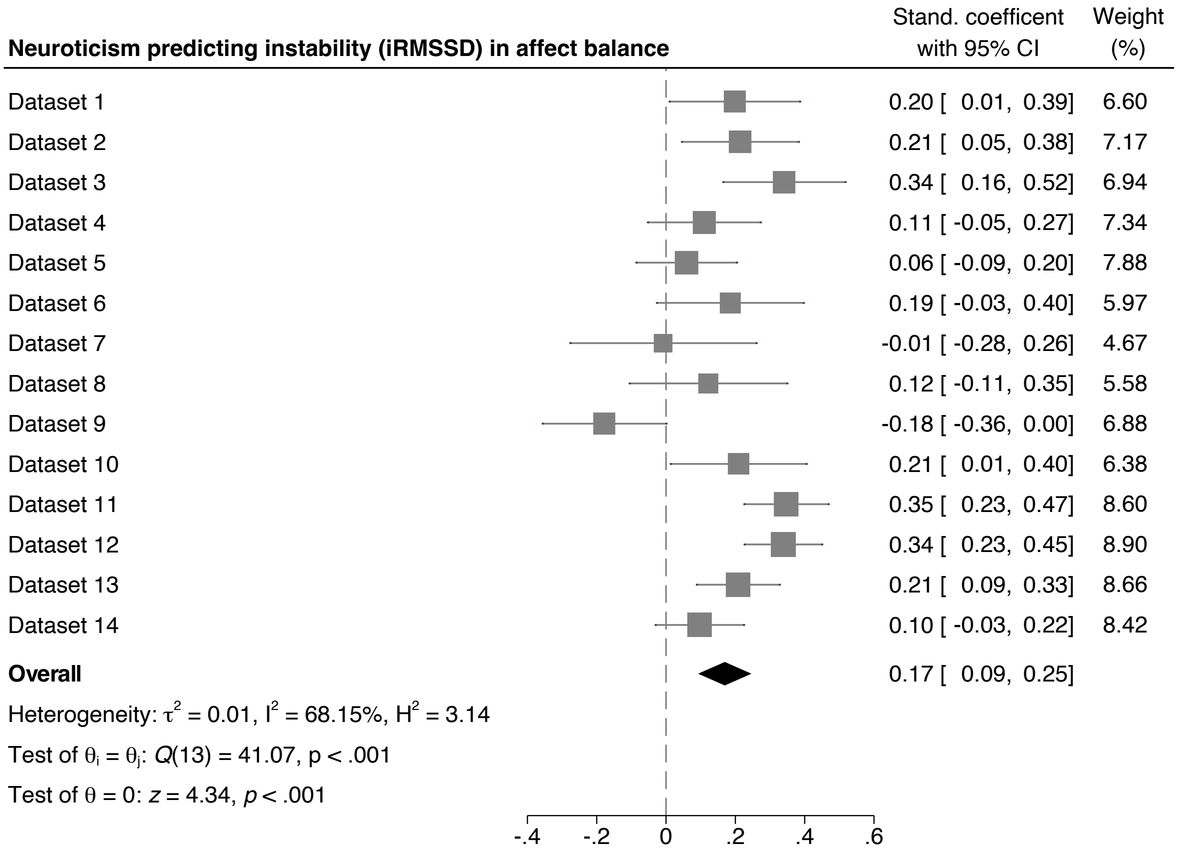

Note. Stand. = standardized.

Figure S6

Forest plot of neuroticism predicting instability in positive affect

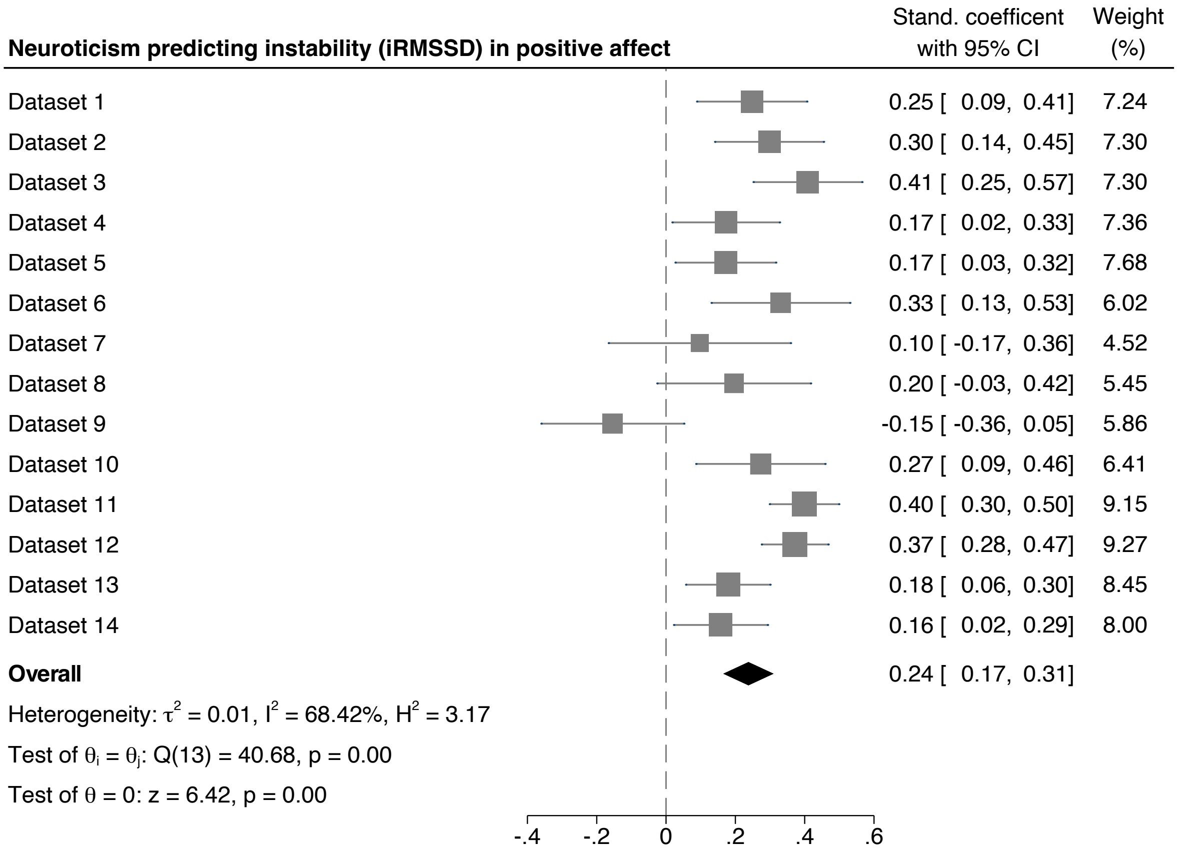

Note. Stand. = standardized.

Figure S7

Forest plot of neuroticism predicting instability in negative affect

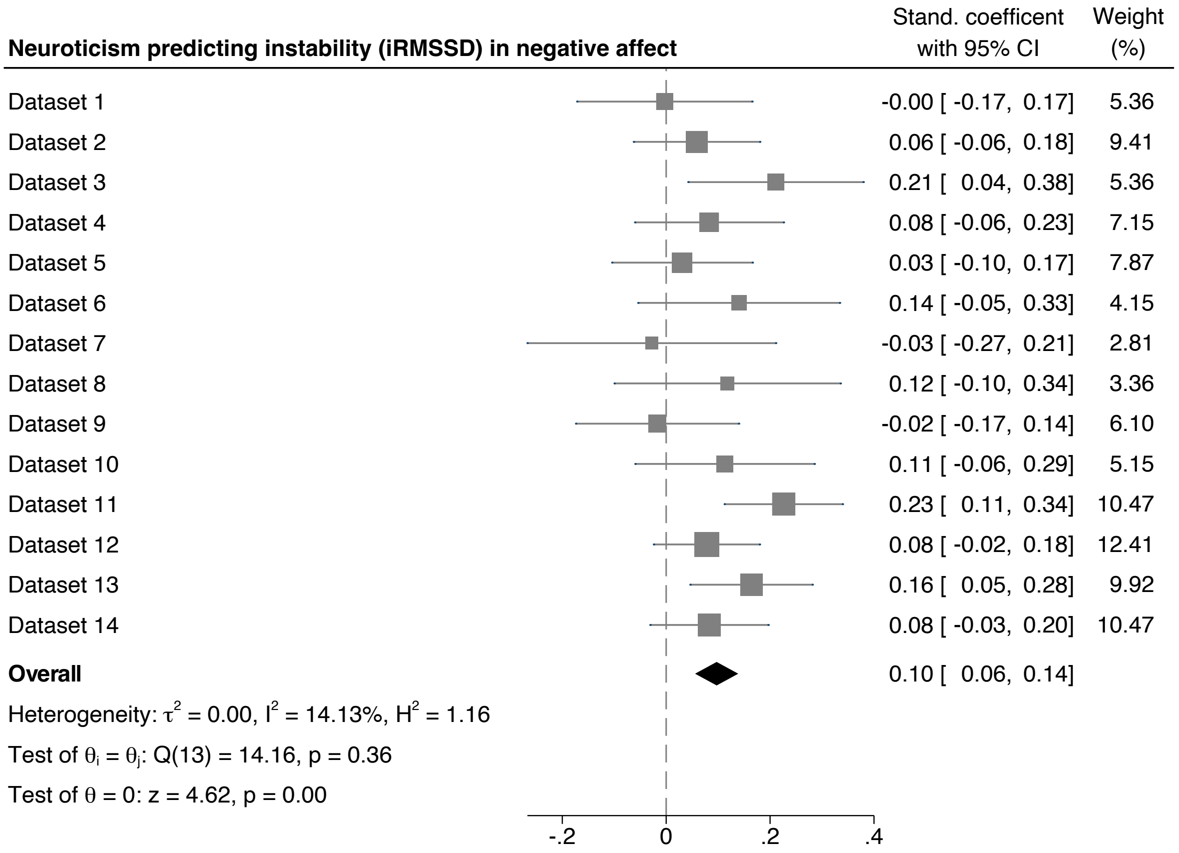

Note. Stand. = standardized.

References

Brans, K., Koval, P., Verduyn, P., Lim, Y. L., & Kuppens, P. (2013). The regulation of negative and positive affect in daily life. *Emotion*, *13*(5), 926–939. https://doi.org/10.1037/a0032400

Dejonckheere, E., Kalokerinos, E. K., Bastian, B., & Kuppens, P. (2019). Poor emotion regulation ability mediates the link between depressive symptoms and affective bipolarity. *Cognition & Emotion*, *33*(5), 1076–1083. https://doi.org/10.1080/02699931.2018.1524747

Erbas, Y., Ceulemans, E., Kalokerinos, E. K., Houben, M., Koval, P., Pe, M. L., & Kuppens, P. (2018). Why I don't always know what I'm feeling: The role of stress in within-person fluctuations in emotion differentiation. *Journal of Personality and Social Psychology*, *115*(2), 179–191. https://doi.org/10.1037/pspa0000126

Grommisch, G., Koval, P., Hinton, J. D. X., Gleeson, J., Hollenstein, T., Kuppens, P., & Lischetzke, T. (2020). Modeling individual differences in emotion regulation repertoire in daily life with multilevel latent profile analysis. *Emotion*, *20*(8), 1462–1474. https://doi.org/10.1037/emo0000669

Holland, E., Koval, P., Stratemeyer, M., Thomson, F., & Haslam, N. (2017). Sexual objectification in women's daily lives: A smartphone ecological momentary assessment study. *The British Journal of Social Psychology*, *56*(2), 314–333. https://doi.org/10.1111/bjso.12152

Koval, P., Holland, E., Zyphur, M. J., Stratemeyer, M., Knight, J. M., Bailen, N. H., Thompson, R. J., Roberts, T.-A., & Haslam, N. (2019). How does it feel to be treated like an object? Direct and indirect effects of exposure to sexual objectification on women's emotions in daily life. *Journal of Personality and Social Psychology*, *116*(6), 885–898. https://doi.org/10.1037/pspa0000161

Mõttus, R., Wood, D., Condon, D. M., Back, M. D., Baumert, A., Costantini, G., Epskamp, S., Greiff, S., Johnson, W., Lukaszewski, A., Murray, A., Revelle, W., Wright, A. G. C., Yarkoni, T., Ziegler, M., & Zimmermann, J. (2020). Descriptive, Predictive and Explanatory Personality Research: Different Goals, Different Approaches, but a Shared Need to Move beyond the Big Few Traits. *European Journal of Personality*, *34*(6), 1175–1201. https://doi.org/10.1002/per.2311

Ringwald, W. R., Manuck, S. B., Marsland, A. L., & Wright, A. G. C. (2022). Psychometric Evaluation of a Big Five Personality State Scale for Intensive Longitudinal Studies. *Assessment*, *29*(6), 1301–1319. https://doi.org/10.1177/10731911211008254

Ringwald, W. R., & Wright, A. G. C. (2022). *Overcoming the confound of means and variability for measuring everyday emotion dynamics related to neuroticism*. PsyArXiv. https://doi.org/10.31234/osf.io/nxbyd

Rowland, Z., Wenzel, M., & Kubiak, T. (2016). The effects of computer-based mindfulness training on Self-control and Mindfulness within Ambulatorily assessed network Systems across Health-related domains in a healthy student population (SMASH): Study protocol for a randomized controlled trial. *Trials*, *17*(1), 570. https://doi.org/10.1186/s13063-016-1707-4

Sosin, A., Kramer, A. C., & Neubauer, A. (2022). *Week-to-week fluctuations in autonomous study motivation: Links to need fulfillment and affective well-being*. PsyArXiv. https://doi.org/10.31234/osf.io/95qaw

Vize, C. E., Ringwald, W. R., Edershile, E. A., & Wright, A. G. C. (2022). Antagonism in Daily Life: An Exploratory Ecological Momentary Assessment Study. *Clinical Psychological Science*, *10*(1), 90–108. https://doi.org/10.1177/21677026211013507

Wenzel, M., & Kubiak, T. (2020). Neuroticism may reflect emotional variability when correcting for the confound with the mean. *Proceedings of the National Academy of*

Sciences, *117*(52), 32857–32858. https://doi.org/10.1073/pnas.2017910117

Wenzel, M., Rowland, Z., & Kubiak, T. (2022). How much variance can event intensity and emotion regulation strategies explain in momentary affect in daily life? *Emotion*, *22*(8), 1969–1979. https://doi.org/10.1037/emo0000816.

Wenzel, M., Rowland, Z., Mey, L. K., Kurth, K., Tüscher, O., & Kubiak, T. (2023). Variability in negative affect is an important feature of neuroticism above mean negative affect once measurement issues are accounted for. *European Journal of Personality*, *37*(3), 338–351. https://doi.org/10.1177/08902070221089139

Wrzus, C., Luong, G., Wagner, G. G., & Riediger, M. (2021). Longitudinal coupling of momentary stress reactivity and trait neuroticism: Specificity of states, traits, and age period. *Journal of Personality and Social Psychology*, *121*(3), 691–706. https://doi.org/10.1037/pspp0000308

Wrzus, C., & Roberts, B. W. (2017). Processes of Personality Development in Adulthood: The TESSERA Framework. *Personality and Social Psychology Review*, *21*(3), 253–277. https://doi.org/10.1177/1088868316652279

Yang, J., Mao, Y., Niu, Y., Wei, D., Wang, X., & Qiu, J. (2020). Individual differences in neuroticism personality trait in emotion regulation. *Journal of Affective Disorders*, *265*, 468–474. https://doi.org/10.1016/j.jad.2020.01.086
